# Contrasting association of Leptin receptor polymorphisms and haplotypes with polycystic ovary syndrome in Bahraini and Tunisian women: a case–control study

**DOI:** 10.1042/BSR20202726

**Published:** 2021-01-06

**Authors:** Meriem Dallel, Zeineb Douma, Ramzi R. Finan, Feten Hachani, Dhafer B. Letaifa, Touhami Mahjoub, Wassim Y. Almawi

**Affiliations:** 1 Laboratory of Human Genome and Multifactorial Diseases (LR12ES07), Faculty of Pharmacy of Monastir, University of Monastir, Monastir, Tunisia; 2 Department of Obstetrics and Gynecology, Hôtel Dieu de France, CHU Université St. Joseph, Beirut, Lebanon; 3 Department of Obstetrics and Gynecology, CHU Farhat Hached, Sousse, Tunisia; 4 Department of Obstetrics and Gynecology, Faculty of Medicine, University of Sousse, Tunisia; 5 Faculty of Sciences, El-Manar University, Tunis, Tunisia; 6 College of Health Sciences, Abu Dhabi University, Abu Dhabi, United Arab Emirates

**Keywords:** Gene association, Genotyping, Leptin receptor, Polycystic ovary syndrome

## Abstract

**Background**: The present study examined the contribution of ethnicity to the association of leptin receptor gene (*LEPR*) gene variants with polycystic ovary syndrome (PCOS) in Tunisian and Bahraini Arabic-speaking women.

**Methods**: Subjects consisted of 320 women with PCOS, and 446 eumenorrhic women from Tunisia, and 242 women with PCOS and 238 controls from Bahrain. Genotyping of (exonic) rs1137100 and rs1137101 and (intronic) rs2025804 *LEPR* variants was done by allelic exclusion.

**Results**: The minor allele frequencies (MAFs) of rs1137100 and rs1137101 were significantly different between PCOS cases and control women from Bahrain but not Tunisia, and *LEPR* rs1137101 was associated with increased PCOS susceptibility only in Bahraini subjects. Furthermore, rs1137100 was associated with decreased PCOS risk among Bahrainis under codominant and recessive models; rs1137100 was negatively associated with PCOS in Tunisians after controlling for testosterone. In addition, rs2025804 was associated with increased PCOS risk among Tunisian but not Bahraini women, after adjusting for key covariates. Negative correlation was seen between rs1137101 and triglycerides in Tunisians, while homeostasis model assessment of insulin resistance (HOMA-IR) and insulin correlated with rs2025804 and rs1137101 among Bahraini subjects, and rs1137101 correlated with estradiol and prolactin. Taking TAG haplotype as common, positive association of TAA and negative association of TGG haplotype with PCOS was seen among Bahraini women; no three-locus PCOS-associated haplotypes were found in Tunisians.

**Conclusions**: The present study is the first to demonstrate the contribution of ethnicity to the association of *LEPR* gene variants with PCOS, thereby highlighting the significance of controlling for ethnicity in gene association investigations.

## Background

Polycystic ovary syndrome (PCOS) ranks as one of the most prevalent endocrine disorders in reproductive age women, with 7–12% prevalence rates reported globally in premenopausal women [1]. As a significant cause of infertility in females, PCOS is defined by the presence of two or more of the following features: chronic oligo-ovulation or anovulation, androgen excess, and polycystic ovaries [2–4]. PCOS is also associated with hormonal disturbances and altered metabolism exacerbated by obesity. These include hyperinsulinemia and insulin resistance (IR), altered glucose homeostasis, and hyperandrogenemia [1,5]. PCOS is a complex disorder with a poorly understood etiology, and several factors were reported to contribute to its pathogenesis [1,3,6]. In particular, genetic factors [7], along with lifestyle and environmental factors, notably high body mass index (BMI), IR, and age at menopause, were identified as contributing risk factors to PCOS development [5,6].

Results of earlier genome-wide association studies (GWASs) on Han Chinese women [8,9] and women of European ancestry [10] identified specific genetic loci linked with PCOS. While not included in the loci identified in the studies of Shi et al. [9] or Day et al. [10], the earlier study of Chen et al. identified single nucleotide polymorphisms (SNPs) mapping to the leptin receptor (LEPR) gene to be associated with PCOS [8], indicating contribution of LEPR to PCOS susceptibility. Encoded by *LEPR* gene, LEPR, also known as obesity receptor (OB-R), is a single *trans*-membrane receptor belonging to the cytokine receptor family [11]. Altered splicing of *LEPR* RNA yields six isoforms, which are grouped into long (LeprB), short (LeprA), and secretory (sOB-R) classes. The expression of *LEPR* gene is selectively localized to the hypothalamus, and to a lower extent in peripheral tissues, including ovaries [12–14], and acts by binding leptin [15,16]. Both LEPR long-form and short-form are involved in the control of thirst and hunger, and influences food intake and energy homeostasis, as well as sleep and body temperature [12,15].

*LEPR* gene is located on chromosome 1p31, and several *LEPR* polymorphisms were identified in genetic association studies. Of these, the two nonsynonymous SNPs (exon 2) Lys^109^Arg (rs1137100) and (exon 4) Gln^223^Arg (rs1137101) [17,18] were linked with metabolic disorders, including obesity [19,20], IR [21], and type 2 diabetes [17,22,23]. Few studies that addressed the association of *LEPR* rs1137100 and rs1137101 variants with PCOS showed inconsistent results [19,23–25]. This was due to differences in ethnic background and also the small sample sizes in some of these studies. Apart from a lone report documenting association of the intronic rs2025804 with lower energy expenditure among Pima Indians [26], no related data on the association of this *LEPR* variant with PCOS were reported.

The present study examined the association of the exonic rs1137100 (Lys^109^Arg) and rs1137101 (Gln^223^Arg), and the intronic rs2025804 *LEPR* polymorphism with PCOS among Bahraini and Tunisian women. Using a larger sample size than that tested previously, the present study is the first to demosntrate contribution of ethnicity in the association of *LEPR* gene variants with PCOS in two distinct Arabic-speaking populations, and the first to identify specific *LEPR* haplotypes linked with altered PCOS risk.

## Methods

### Study population

This retrospective population-based case–control study was carried out at the OB/GYN outpatient clinics of Hôpital Frahat Hached (Sousse, Tunisia) and Salmaniya Medical Complex (Manama, Bahrain). Between January 2012 and December 2015, 320 Tunisian and 242 Bahraini unrelated PCOS cases were enrolled into the study. The diagnosis of PCOS was based on the 2003 Rotterdam Criteria [25], in which two of the three conditions needed confirmation: oligo-ovulation or anovulation, ultrasound evidence of polycystic ovaries, and clinical and/or biochemical hyperandrogenism.

Cases were excluded if they presented with hyperandrogenism of unrelated causes, active thyroid disease, and hyperprolactinemia. They were also excluded if they presented with extremes of BMI [calculated as: weight (kg)/height (m^2^)], namely <18 or >50 kg/m^2^, or if they were on medication that affected gonadal function (hormonal contraceptives, clomiphene), or glucose homeostasis (metformin or thiazolidinediones) for at least 6 months (anti-androgenic) or 1 month (insulin sensitizers) prior to recruitment into the study. All PCOS cases underwent transvaginal ultrasound scanning for assessing ovarian morphology; polycystic ovaries defined as ovaries with ≥12 subcapsular follicles of 2–9 mm in diameter, or ovaries with total volumes exceeding 10 cm^3^. Modified Ferriman–Gallwey (m-FG) scoring was used for assessment of hirsutism, which was confirmed if the score was ≥6.

The control group comprised 446 Tunisian and 238 Bahraini eumenorrheic women, with no evidence of present or past obstetric-gynecological complications, or under any medical treatment. While Bahraini cases and control were age-matched (*P*=0.06), the mean age of cases (30.9 ± 4.7 years) was lower than that of controls (31.8 ± 6.0 years) (*P*=0.02) (Table 1). On all PCOS cases and control women, we collected demographic and biochemical data, and personal and family history of hypertension, hyperlipidemia, and diabetes. Study participants consented in writing to participate in the study, which was granted approval by research and ethics committees of Hôpital Frahat Hached (number: RE/TM/04-100; awarded 12 November 2011) in Tunisia, and Salmaniya Medical Complex in Bahrain (number: SMC/Research/No. 145/2012, awarded 27 January 2012).

**Table 1:** Demographic and clinical characteristics of study subjects

	Bahraini			Tunisian		
Parameter	Cases	Controls	*P*^1^	Cases	Controls	*P*^1^
Number	242	238		320	446	
Age (years)^2^	28.6 ± 6.0	27.4 ± 7.1	0.06	30.9 ± 4.7	31.8 ± 6.0	0.02
BMI (kg/m^2^)	29.2 ± 6.6	26.2 ± 5.3	<0.001	29.1 ± 5.5	25.9 ± 5.4	<0.001
Waist-hip ratio	0.9 ± 0.1	0.9 ± 0.1	0.41	0.9 ± 0.1	0.9 ± 0.05	0.22
LH/FSH^ 4^	1.6 ± 1.0	1.5 ± 1.4	0.79	1.4 ± 0.8	1.3 ± 0.6	0.08
Testosterone (nmol/l)^3^	2.4 (0.3−15.1)	1.6 (0.3−5.1)	0.011	2.6 (0.2−12.4)	1.0 (0.4−5.3)	<0.001
SHBG ^4^, nmol/l	25.3 (10.8−134.0)	55.2 (12.6−224.4)	<0.001	29.7 (12.7−290.6)	46.0 (5.2−165.3)	0.01
Insulin, mIU/l	10.31 (1.5−99.3)	6.43 (1.7−81.7)	0.001	10.9 (0.75−80.2)	7.4 (1.2−53.0)	0.02
HOMA-IR	2.7 (0.3−56.1)	1.5 (0.3−18.5)	<0.001	3.2 (0.2−43.7)	1.3 (0.02−16.2)	<0.001
TG ^4^, mmol/l	1.4 ± 0.9	1.1 ± 0.7	0.06	1.7 ± 1.0	1.3 ± 0.8	0.07
LDL-cholesterol ^4^	3.9 ± 0.3	3.8 ± 0.1	0.99	4.6 ± 1.4	3.9 ± 1.4	0.03

1 Student’s *t* test (variable with normal distribution), Mann–Whitney U test (variables that were not normally distributed).

2 Mean ± SD.

3 Median (range).

Abbreviations: FSH, follicle-stimulating hormone; LDL, low-density lipoprotein; LH, leutinizing hormone; SHBG, sex hormone binding globulin; TG, triglycerides.

### Biochemical analysis

Peripheral venous blood samples were obtained at 7:00–9:00 a.m. during the early follicular phase of the menstrual cycle (days 2–5) for control subjects, or any day for women with PCOS, after an overnight (>12 h) fast. FSH, LH, and total testosterone were determined using immunofluorometric assay or radioimmunoassay (coefficients of variation (CVs) < 5%; intra-day precision, 2.4–3.1%; inter-day precision, 7.1–7.9%, for all tests). Insulin was measured by enzyme-linked immunosorbent assay (ELISA) according to manufacturer’s instructions (R&D Systems, Minneapolis, MN). Homeostasis model assessment of IR (HOMA-IR) was calculated as: fasting glucose (mmol/l)/fasting insulin (mIU/ml))/22.5, used as cases and controls spanned several BMI categories lower limit of quantitation.

### LEPR genotyping

We selected three SNPs in *LEPR* gene with minor allele frequencies (MAFs) of 5% or higher, and with reported clinical relevance, as noted from NCBI Geneview. The selected variants were (intron 2) rs2025804), and the missense variants rs1137100 (Lys^109^Arg; exon 4) and rs1137101 (Gln^223^Arg; exon 6). Genotyping of the studied *LEPR* gene variants was done by allelic discrimination, using VIC- and FAM-labeled probes. TaqMan assays for rs20258004 (C__12111710_20), rs1137100 (C____518168_20), and rs1137101 (C___8722581_10) were purchased from Applied Biosystems (ABI Thermo Fisher, Foster City, CA).

Genotyping was done in 6-μl reaction volume on StepOne Plus and 7500 real-time PCR systems, according to the specific instructions of the manufacturer (ABI Thermo Fisher). The reproducibility of genotyping was assessed through retesting blinded control and case samples; the concordance consistently exceeded 99%. The genotype frequencies of the tested *LEPR* SNPs were comparable with those found in HapMap CEU database and were in Hardy–Weinberg equilibrium (HWE).

### Statistical analysis

SPSS v. 24 (IBM; Armonk, NY) was employed for analyzing the results, which were presented as means and ± SD (normally distributed continuous data), or percent total (categorical parameters). Student’s *t* test and Pearson’s χ^2^ test were used for assessing mean differences in or differences in proportions, respectively. All analyses were conducted under the additive genetic model using SNPStats (bioinfo.iconcologia.net/snpstats/). Haploview 4.2 (http://broad.mit.edu/mpg/haploview) was utilized for HWE assessment of the distribution of *LEPR* genotypes in control women, and calculation of the study power was determined using GAS Power Calculator (http://csg.sph.umich.edu/abecasis/gas_power_calculator/index.html). After considering sample size (cases and controls), disease allele frequency, and genotype relative risk, and significance level, the overall study power (average of three variants) was calculated at 0.680 (Tunisians) and 0.798 (Bahraini).

Haploview 4.2 was used for determination of the extent of linkage disequilibrium (LD) between combination of *LEPR* pair of SNPs. This was followed by the construction of three-locus haplotypes by the expectation maximization method, using the default method of Gabriel et al. [27]. Multiple comparisons were corrected by Bonferroni method, according to: corrected *P* (*Pc*) = 1− [1 − *P*)^*n*^]; *n* denoting number of comparisons. Odds ratios (ORs) and 95% confidence intervals (CIs) for the association between *LEPR* SNPs and PCOS were determined using logistic regression analyses; *P*<0.05 was considered statistically significant.

## Results

### Study subjects

Table 1 presents the demographic and clinical characteristics of Bahraini (242 and 238) and Tunisian (320 and 446) PCOS cases and control subjects. Significantly higher BMI was recorded for both Tunisian (*P*<0.001) and Bahraini (*P*<0.001) PCOS cases when compared with control women. Lower serum SHBG (*P*=0.01 in Tunisians; *P*<0.001 in Bahraini), higher fasting serum insulin (*P*=0.02 in Tunisians; *P*=0.001 in Bahraini), HOMA-IR (*P*<0.001 in both Tunisians and Bahraini), and total testosterone (*P*=0.011 in Bahraini; *P*<0.001 in Tunisians), were also seen in women with PCOS and control women. Furthermore, mean age at blood sampling (*P*=0.02), and LDL cholesterol (*P*=0.03) were significantly different between Tunisian PCOS cases and control women, but not Bahraini cases and control subjects (*P*=0.06 and *P*=0.99, respectively). Furthermore, LH/FSH ratio was not significantly different between PCOS cases and control women in Bahraini (*P*=0.79) and Tunisian (*P*=0.08) subjects.

### Association studies

The *LEPR* variants tested comprised one intronic (rs2025804) and two exonic missense (rs1137100 and rs1137101) variants. Table 2 lists the characteristics of the three genotyped *LEPR* SNPs. Marginal departure from HWE was noted for rs1137100 in Bahraini (*P*=0.04) and rs1137101 in Tunisians (*P*=0.01). The distribution of the minor alleles of rs1137100, rs1137101, and rs2025804 in Tunisian and Bahraini women with PCOS and control women are shown in Table 3. Significantly lower rs1137100 (*P*=0.008) and higher rs1137101 (*P*<0.001) MAF was seen in Bahraini women with PCOS compared with ethnically matched controls, which persisted after the Bonferroni correction for multiple testing. In contrast, no statistically significance difference in the MAF of the three tested variants was seen between Tunisian PCOS cases and control subjects.

**Table 2:** *LEPR* SNPs analyzed

SNP	Position^1^	Location	Allele	Assay ID	HWE (Bahrain)	HWE (Tunisian)
rs2025804	65480438	Intron	T>C	C__12111710_20	0.78	0.08
rs1137100	65570758	Lys109Arg	A>G	C____518168_20	0.04	0.24
rs1137101	65592830	Gln223Arg	A>G	C___8722581_10	0.09	0.01

1 Location on chromosome 1 (GRCh38.p12).

**Table 3:** Association of *LEPR* SNPs with PCOS in Bahraini and Tunisian subjects

	Bahraini^1^							Tunisians^1^				
SNP	% Genotyped	Cases^2^	Controls^2^	χ^2^	*P*^3^	OR (95% CI)	% Genotyped	Cases	Controls	χ^2^	P^2^	OR (95% CI)
rs2025804	96.3	0.208	0.230	0.62	0.431	0.88 (0.63–1.22)	97.4	0.161	0.171	0.25	0.617	0.93 (0.69–1.24)
rs1137100	98.4	**0.143**	**0.212**	**7.00**	**0.008**	**0.62 (0.43–0.89)**	98.6	0.118	0.138	1.17	0.323	0.84 (0.61–1.16)
rs1137101	97.4	**0.382**	**0.252**	**16.20**	**5.7 × 10^−5^**	**1.83 (1.36– 2.46)**	98.3	0.415	0.411	0.03	0.689	1.02 (0.82–1.27)

**Boldface** indicates statistically significant differences.

1 Study subjects (case/control) were: Bahraini (242/238) and Tunisians (320/446).

2 Minor allele frequency.

3 *P*-value adjusted for age and BMI.

Comparable genotype distribution of the three tested variants was seen in Tunisian women with PCOS and control women (Table 4). In contrast, significant differences in the genotype distribution of rs1137100 (*P*=0.009) favoring a protective nature of rs1137100, and rs1137101 (*P*=0.001) favoring an at-risk nature of this variant, were seen in Bahraini subjects (Table 4). Similar to their allelic distribution, this indicates that the association of the tested *LEPR* variants with PCOS is dependent on the ethnic background of the studied population.

**Table 4:** Distribution of *LEPR* genotypes in Bahraini and Tunisian PCOS cases and control subjects

	Bahraini								Tunisian							
	1/1^1^		1/2		2/2				1/1		1/2		2/2			
SNP	Cases	Controls	Cases	Controls	Cases	Controls	χ^2^	*P*	Cases	Controls	Cases	Controls	Cases	Controls	χ^2^	*P*
rs2025804	0.64^2^	0.59	0.31	0.36	0.05	0.05	1.17	0.56	0.71	0.71	0.25	0.24	0.04	0.05	0.70	0.71
rs1137100	**0.73**	**0.66**	**0.25**	**0.27**	**0.02**	**0.08**	**9.46**	**0.009**	0.77	0.76	0.22	0.21	0.01	0.03	3.54	0.17
rs1137101	**0.41**	**0.59**	**0.41**	**0.31**	**0.18**	**0.10**	**14.58**	**0.001**	0.35	0.37	0.47	0.44	0.18	0.19	0.20	0.91

**Boldface** indicates statistically significant differences.

1 Genotypes were coded as: 1, major allele; 2, minor allele.

2 Genotype frequencies.

As shown in Table 5, rs1137100 in the LEPR gene was associated with a 0.65‐fold reduced risk of PCOS in the log‐additive model (OR = 0.65, 95% CI = 0.46–0.91, *P*=0.01), and with 0.02-fold (OR = 0.02, 95% CI = 0.07–0.62, *P*<0.001) and 0.21-fold (OR = 0.21, 95% CI = 0.07–0.65, *P*<0.001) in the codominant and recessive (G/G vs. A/A+A/G) models, respectively. This association was lost when examined under the dominant (A/A vs. A/G+G/G) model (OR = 0.70, 95% CI = 0.46–1.05, *P*=0.08). Except for marginal association under the recessive model (OR = 0.32, 95% CI = 0.09–1.11, *P*=0.05), no association of the rs1137100 with PCOS was seen under the remaining model. Likewise, rs1137101 was associated with increased risk of PCOS when examined under log‐additive (OR = 1.69, 95% CI = 1.28–2.24, *P*<0.001), codominant (OR = 1.92, 95% CI = 1.26–2.93, *P*<0.001), dominant (OR = 2.08, 95% CI = 1.41–3.07, *P*<0.001), and recessive (OR = 1.96, 95% CI = 1.10–3.51, *P*=0.02) genetic models (Table 5).

**Table 5:** Association of *LEPR* genotypes with PCOS according to different genetic models

		Bahraini		Tunisian	
SNP	Model^1^	*P*	OR (95% CI)	*P*	OR (95% CI)
rs2025804	Additive	0.43	0.88 (0.63–122)	0.72	0.95 (0.72–1.25)
	Codominant	0.56	0.8 (0.53–1.21)	0.68	1.05 (0.73–1.49)
	Dominant	0.32	0.82 (0.55–1.22)	0.96	0.99 (0.71–1.39)
	Recessive	0.91	1.05 (0.44–2.53)	0.40	0.72 (0.33–1.55)
rs1137100	Additive	**0.01**	**0.65 (0.46–0.91)**	0.36	0.87 (0.63–1.18)
	Codominant	**6.9 × 10^−3^**	**0.20 (0.07–0.62)**	0.14	1.03 (0.71–1.49)
	Dominant	0.08	0.70 (0.46–1.05)	0.71	0.94 (0.65–1.34)
	Recessive	**2.2 × 10^−3^**	**0.21 (0.07–0.65)**	**0.05**	0.32 (0.09–1.11)
rs1137101	Additive	**2.0 × 10^−4^**	**1.69 (1.28–2.24)**	0.92	1.01 (0.82–1.25)
	Codominant	**6.1 × 10^−4^**	**1.92 (1.26–2.93)**	0.85	1.09 (0.78–1.53)
	Dominant	**2.0 × 10^−4^**	**2.08 (1.41–3.07)**	0.71	1.06 (0.77–1.46)
	Recessive	**0.02**	**1.96 (1.10–3.51)**	0.79	0.95 (0.64–1.40)

**Boldface** indicates statistically significant differences.

1 Determined by SNPstats (http://bioinfo.iconcologia.net/snpstats/start.htm).

### Correlation between *LEPR* variants and PCOS phenotypic features

We examined the correlation between each *LEPR* variant in Tunisians and Bahrainis and the continuous parameters age, BMI, fasting serum insulin and serum glucose, HOMA-IR, total testosterone, serum SHBG, along with total cholesterol and triglycerides. The correlation index (r) and *P*-value are shown in Table 6. Positive correlation between rs2025804 and SHBG (r = 0.265, *P*=0.044), and negative correlation between rs1137101 and serum triglycerides (r = −0.202, *P*=0.016) was found in Tunisians. While not associated with PCOS in Bahrainis *per se*, positive correlation was noted between rs2025804 and fasting insulin (r = 0.110, *P*=0.049) and with HOMA-IR (r = 0.122, *P*=0.032). Moreover, positive correlation was also found between rs1137100 and SHBG (r = 0.202, *P*=0.001), and between rs1137101 and insulin (r = 0.162, *P*=0.003), and with HOMA-IR (r = 0.146, *P*=0.010).

**Table 6:** Correlation between *LEPR* variants and PCOS phenotypic features in Bahraini and Tunisian subjects

	Tunisian						Bahraini					
Parameter	rs2025804		rs1137100		rs1137101		rs2025804		rs1137100		rs1137101	
	*r*	*P*	*R*	*P*	*r*	*P*	*r*	*P*	*r*	*P*	*r*	*P*
**Age**	0.028	0.518	−0.026	0.539	−0.051	0.228	0.055	0.293	−0.084	0.10	0.086	0.095
**BMI**	−0.021	0.610	−0.06	0.150	−0.054	0.195	0.045	0.421	0.003	0.963	0.044	0.430
**Insulin**	0.208	0.102	−0.035	0.785	−0.093	0.473	0.110	**0.049**	0.025	0.654	0.162	**0.003**
**GLU**	−0.055	0.510	−0.128	0.127	0.003	0.976	0.01	0.849	0.054	0.306	0.017	0.756
**HOMA.IR**	0.201	0.150	0.02	0.891	−0.029	0.841	0.122	**0.032**	−0.002	0.974	0.146	**0.010**
**TG**	−0.071	0.395	−0.116	0.167	−0.202	**0.016**	0.083	0.321	0.008	0.924	0.023	0.782
**Cholesterol**	−0.055	0.514	−0.068	0.416	0.086	0.310	0.026	0.755	−0.011	0.893	−0.092	0.267
**TT**	−0.002	0.988	0.141	0.190	−0.002	0.983	−0.037	0.563	0.038	0.544	0.033	0.608
**SHBG**	0.265	**0.044**	0.111	0.401	−0.005	0.973	0.059	0.324	0.202	**0.001**	−0.06	0.318

**Boldface** indicates statistical significance.

### Haploview analysis

Moderate LD was found among the three tested *LEPR* SNPs between the two studied populations, which allowed construction of three-locus haplotypes. All haplotypes (100.0%) were captured by the maximum eight haplotypes in Bahrainis, while all haplotypes among Tunisians were captured by only seven. Taking TAA haplotype as common (OR = 1.00) for both populations, strong positive association of TAG (*P*<0.001), and significantly negative association of TGG (*P*<0.001), and CGA (*P*=0.038) haplotypes with PCOS were seen among Bahraini women (Table 7). These differences remained significant after correcting for multiple comparisons in the case of TAG haplotype (*Pc* = 7.8 × 10^−5^) and TGG (*Pc* = 5.8 × 10^−4^), but not CGA (*Pc* = 0.266) haplotypes. In contrast, no PCOS-associated three-locus haplotypes were identified in Tunisians, consistent with the differences between the two populations.

**Table 7:** Association of *LEPR* haplotypes with PCOS in Bahraini and Tunisian subjects

	Bahraini					Tunisian				
Haplotype	Total	Cases	Controls	χ^2^	*P*	Total	Cases	Controls	χ^2^	*P*
T A A	0546	0.529	0.564	1.057	0.304	0.526	0.529	0.524	0.035	0.852
T A **G**	0.174	0.229	0.115	19.559	9.75 × 10^−6^	0.266	0.279	0.258	0.745	0.388
**C G G**	0.096	0.101	0.091	0.244	0.621	0.087	0.087	0.086	0.007	0.935
**C** A A	0.079	0.070	0.090	1.165	0.281	0.053	0.047	0.057	0.727	0.394
T **G** A	0.041	0.015	0.068	15.733	7.29 × 10^−5^	0.008	0.006	0.010	0.650	0.420
**C** A **G**	0.026	0.028	0.023	0.298	0.585	0.023	0.023	0.023	0.012	0.912
T **G G**	0.023	0.021	0.026	0.192	0.662	0.037	0.029	0.042	1.539	0.215
**C G** A	0.015	0.007	0.024	4.297	0.038					

1 LEP three-locus haplotype (rs2025804, rs1137100, rs1137101); boldface and underlined indicate minor allele.

## Discussion

The association of *LEPR* gene with pathologies, including obesity [20], diabetes [17,22], pregnancy complications [28–30], cancer [31,32] was investigated in several populations. Few studies on Caucasian and Asian populations examined the association of *LEPR* genetic variants with PCOS, but with inconclusive findings, and an ethnic contribution to this association was suggested. This was exemplified by the association of rs1137100 and rs1137101 with PCOS in Chinese [23,24] and Koreans [33], but not in Caucasians [19,25]. We recently reported on the differential association of *LEP* gene variants and haplotypes with PCOS in Tunisian and Bahraini subjects [34]. Here, we extend these findings by demonstrating differential association of rs1137100 and rs1137101 with PCOS in Bahraini, and rs2025804 in Tunisians. This confirms the contribution of ethnicity to the association of *LEP* and *LEPR* variants with PCOS.

This is the first study to examine the association of *LEPR* rs1137100, rs1137101, and rs2025804 genetic variants with PCOS in Middle Eastern-North African Arabic-speaking populations. Arabs comprise diverse ethnic populations that extend from the Arabian (Persian) Gulf to the Atlantic Ocean. In view of the contribution of ethnicity to genetic association studies, coupled with the differences in the distribution of specific gene variants in different racial/ethnic populations, we investigated the association of *LEPR* polymorphic variants with PCOS in individuals from two distinct Arab communities: Tunisia (North Africa) and Bahrain (Arabian Peninsula). Both PCOS cases and control women were self-declared Tunisian and Bahraini Arabs and were consecutively enrolled. Unlike previous studies on Caucasian populations which involved low sample size [19,25], our studies included a larger sample of each population, with estimated power of 74%, according to the sample size in Bahraini (242 cases, 238 controls) and Tunisian (320 cases, 446 controls), and the prevalence of PCOS in each community.

Contrasting associations of rs1137100 and rs1137101 *LEPR* variants with PCOS was noted among Bahraini women, highlighted by the negative association of rs1137100 and positive association of rs1137101 with PCOS. This imparted protective and at-risk nature to these variants, respectively. No association of either variant with PCOS was seen in Tunisian women. As for the rs1137100 (Lys^109^Arg, K109R) variant, our results are in accord with a recent Chinese study involving 326 cases and 283 control women, in which the minor allele of rs1137100 was described as ‘protective’ of PCOS [23], but in apparent disagreement with an earlier Finnish study involving 36 PCOS cases and 122 control subjects [25], which reported lack of association of rs1137100 with PCOS. These apparent inconsistencies can be reconciled by differences in ethnicity and sample size.

The rs1137101 (Gln^223^Arg, Q223R) was positively associated with PCOS in Bahraini, but not Tunisian women. This was in contrast with a Korean study involving 229 PCOS cases and 150 eumenorrheic women, in which rs1137101 was negatively associated with PCOS (OR = 0.446, *P*<0.001) [33]. Furthermore, Chinese [23], Saudi [19], and Finnish [25] studies reported no association of rs1137101 with PCOS. The low sample size in Oksanen et al. [24] and Daghestani et al. [18] studies, and ethnic differences between our study subjects and Asian studies [23,33] warrant careful assignment of the nature of the association of this variant with PCOS.

In addition to the two nonsynonymous polymorphisms, we tested the association of the intronic rs2025804 with PCOS. These variants were associated with lower 24-h energy expenditure in Pima Indians [25], and with heightened inflammatory responses accompanying anti-tuberculosis drug-induced liver injury in Chinese patients [35]. While it was associated with PCOS *per se*, carriage of rs2025804 minor allele correlated with IR in Bahraini PCOS cases, and with altered SHBG levels in Tunisian subjects. Further studies on different populations are needed to confirm, or alternatively rule out the association of this variant with PCOS and associated features.

Functionally, the Lys^109^Arg (rs1137100) and Gln^223^Arg (rs1137101) mutations are localized in the extracellular domain of LEPR, and directly affect the binding of leptin by altering the three-dimensional conformation of LEPR, and consequently downstream LEPR-induced sinaling events. The differential association of these variants with PCOS in Bahraini and Tunisian women is reminiscent of the findings of a recent meta-analysis involving 33 studies, which also reported on the ethnicity dependency of the association of rs1137101 with diabetes [36].

In conclusion, we documented an association between rs1137100 and rs1137101 *LEPR* gene polymorphisms and PCOS in Bahraini Arab women, with a possible protective and at-risk nature imparted to rs1137100, and rs1137101, respectively. No similar association was seen in Tunisian Arab women. Our study has some strengths. The studied populations were ethnically homogeneous, and the study was sufficiently powered. There were also limitations in the present study, namely the inclusion of three *LEPR* variants, which raises the possibility of association of additional *LEPR* variants in PCOS in both populations. The differential association of the *LEPR* variants with PCOS in Bahraini and Tunisian women is consistent with differences in ethnic background of Bahraini and Tunisian populations, the former deriving their origin from Arabian Peninsula, Iraq, Iran, and possibly East Africa, while the latter derive their origin from East Mediterranean, North African, Turkish, and European origins. The retrospective case–control study design is another limiting factor toward addressing the possible cause–effect relationship, and the diagnostic/prognostic utility of these genetic variants in PCOS remains to be seen. Despite these shortcomings, our study confirm the dependency of ethnicity to the association of *LEPR* variants with PCOS.

## Data Availability

The data contained in the present study are available at the Dryad Data Repository, and can be accessed at https://datadryad.org/stash/dataset/doi:10.5061/dryad.8467g.

## Abbreviations

BMI: body mass index
CI: confidence interval
HOMA-IR: homeostasis model assessment of IR
HWE: Hardy–Weinberg equilibrium
IR: insulin resistance
LD: linkage disequilibrium
LEPR: leptin receptor
MAF: minor allele frequency
OB-R: obesity receptor
OR: odds ratio
PCOS: polycystic ovary syndrome
SNP: single nucleotide polymorphism
